# Individualized Mutation Detection in Circulating Tumor DNA for Monitoring Colorectal Tumor Burden Using a Cancer-Associated Gene Sequencing Panel

**DOI:** 10.1371/journal.pone.0146275

**Published:** 2016-01-04

**Authors:** Kei A. Sato, Tsuyoshi Hachiya, Takeshi Iwaya, Kohei Kume, Teppei Matsuo, Keisuke Kawasaki, Yukito Abiko, Risaburo Akasaka, Takayuki Matsumoto, Koki Otsuka, Satoshi S. Nishizuka

**Affiliations:** 1 Molecular Therapeutics Laboratory, Department of Surgery, Iwate Medical University School of Medicine, Morioka, Japan; 2 Department of Surgery, Iwate medical University School of Medicine, Morioka, Japan; 3 Division of Biomedical Information Analysis, Iwate Tohoku Medical Megabank Organization, Iwate Medical University, Yahaba, Japan; 4 MIAST (Medical Innovation by Advanced Science and Technology) Project, Iwate Medical University School, Morioka, Japan; 5 Institute of Biomedical Science, Iwate Medical University, Yahaba, Japan; 6 Division of Gastroenterology and Hepatology, Department of Internal Medicine, Iwate Medical University School of Medicine, Morioka, Japan; 7 Department of Surgery, Iwate Medical University School of Dentistry, Morioka, Japan; CNR, ITALY

## Abstract

**Background:**

Circulating tumor DNA (ctDNA) carries information on tumor burden. However, the mutation spectrum is different among tumors. This study was designed to examine the utility of ctDNA for monitoring tumor burden based on an individual mutation profile.

**Methodology:**

DNA was extracted from a total of 176 samples, including pre- and post-operational plasma, primary tumors, and peripheral blood mononuclear cells (PBMC), from 44 individuals with colorectal tumor who underwent curative resection of colorectal tumors, as well as nine healthy individuals. Using a panel of 50 cancer-associated genes, tumor-unique mutations were identified by comparing the single nucleotide variants (SNVs) from tumors and PBMCs with an Ion PGM sequencer. A group of the tumor-unique mutations from individual tumors were designated as individual marker mutations (MMs) to trace tumor burden by ctDNA using droplet digital PCR (ddPCR). From these experiments, three major objectives were assessed: (a) Tumor-unique mutations; (b) mutation spectrum of a tumor; and (c) changes in allele frequency of the MMs in ctDNA after curative resection of the tumor.

**Results:**

A total of 128 gene point mutations were identified in 27 colorectal tumors. Twenty-six genes were mutated in at least 1 sample, while 14 genes were found to be mutated in only 1 sample, respectively. An average of 2.7 genes were mutated per tumor. Subsequently, 24 MMs were selected from SNVs for tumor burden monitoring. Among the MMs found by ddPCR with > 0.1% variant allele frequency in plasma DNA, 100% (8 out of 8) exhibited a decrease in post-operation ctDNA, whereas none of the 16 MMs found by ddPCR with < 0.1% variant allele frequency in plasma DNA showed a decrease.

**Conclusions:**

This panel of 50 cancer-associated genes appeared to be sufficient to identify individual, tumor-unique, mutated ctDNA markers in cancer patients. The MMs showed the clinical utility in monitoring curatively-treated colorectal tumor burden if the allele frequency of MMs in plasma DNA is above 0.1%.

## Introduction

Quantitative assessment of circulating tumor DNA (ctDNA) has been shown to be useful for monitoring tumor burden in response to treatment [1, 2]. However, mutated genes in many types of cancers represent only a few percent of the entire number of genes present, suggesting that only a limited number of genes are associated with cancer development and progression [3, 4]. Therefore, a set of selective genes known to be associated with cancer is fundamentally needed to monitor tumor burden. In fact, monitoring treatment efficacy by ctDNA has been performed using a set of well-studied target genes, including *KRAS*, *BRAF*, *HER2*, and others [5–9]. On the other hand, information on monitoring tumor burden after surgical intervention is limited because it remains unknown which tumor-unique mutated genes should be monitored for each patient [10]. In fact, data have implied that a limited number of tumor-unique mutations may sufficiently represent the volume and characteristics (e.g., drug resistance) of individual tumors [11]. If a small number of tumor-unique mutations are identified from primary tumors, then they could be used to detect the mutations in ctDNA. This represents an advantageous and cost effective approach for monitoring tumor burden after surgical intervention.

The idea of using ctDNA from cancer patients to monitor tumor burden led us to design the current study focused on colorectal cancer patients who had received curative removal of the tumor. Our strategy was to collect individual colorectal tumor samples through endoscopic or laparoscopic colorectal tumor curative resection as well as blood specimens. In contrast to previous studies using extremely advanced tumors, including cases with incomplete resection [1, 2, 7], our results demonstrate that individual marker mutations (MMs) in ctDNA may be useful for monitoring post-operative, resectable colorectal tumor burden on the basis of decreased allele frequency of ctDNA in post-operative plasma.

## Patients and Methods

### Human samples and study design

This study was approved by the Institutional Review Board of Iwate Medical University in compliance with the Helsinki declaration (HG H24-22). An individual written consent was obtained from all participants and all analyses were performed anonymously. In principle, patients were eligible if their surgical or endoscopic resection was indicated for benign or Stage 0 to III colorectal tumors, and had no previous history of any treatment at the time of informed consent. All analyzable cases were required to provide the following four types of materials: pre- and post-operational plasma (at least 24 h after tumor resection), primary tumor, and peripheral blood mononuclear cells (PBMCs). Blood samples were drawn for routine pre- and post-operational laboratory examinations. Either eight or 16 ml of blood was collected in a BD Vacutainer CPT blood collection tube (Becton, Dickinson and Company, East Rutherford, NJ). Within two hours post-collection, the tubes were centrifuged at 1800 *g* for 20 min at room temperature to separate into plasma and PBMC layers. The upper phase of eight ml of blood was then transferred into a five ml tube labeled with the patient-unique identification number. The tubes were immediately stored at -80°C until DNA isolation. Total genomic DNA was extracted using the QIAamp Circulating Nucleic Acid Kit for plasma and the QIAamp DNA Mini Kit for primary tumors and PBMCs (Qiagen, Venlo, The Netherlands). The quantity of extracted DNA was measured using the Qubit® 2.0 dsDNA high sensitivity assay (Life Technologies, Carlsbad, CA). In the present study, our preliminary experiment confirmed that leaving 5-7mm of "buffering" layer from the buffy coat after the centrifuge sufficiently prevents the plasma layer from contamination of blood and cell debris, and yields acceptable DNA quality [12, 13]. Relative copy number of the genome in plasma DNA was also estimated by quantitative-PCR (qPCR) for the LINE-1 gene using the primer sets previously described [14].

### DNA extraction from human colon cancer cell line

The human colon cancer cell line, HCT116, was obtained in 2008 from the Division of Cancer Treatment and Diagnosis Tumor Repository, National Cancer Institute (NCI MTA #1-2093-08). The cell line was cultured in RPMI-1640 supplemented with 10% FBS and the genomic DNA was extracted using a QIAamp DNA Mini Kit (Qiagen, Venlo, The Netherlands) within three passages after thawing.

### Multiplex PCR and library construction using CHPv2

The CHPv2 is a pool of PCR primers that target 207 amplicons for 2885 mutations in 50 cancer-associated genes [15] (Life Technologies, Carlsbad, CA). The entire list of genes is available through the supplier’s website (http://tools.invitrogen.com/downloads/cms_106003.csv). Approximately 10 ng of DNA per sample was used for amplicon production by multiplex PCR using the Ion AmpliSeq CHPv2 and Ion AmpliSeq Library Kit 2.0 (Life Technologies, Carlsbad, CA). The resulting multiplex PCR reaction pool was used for target sequence library preparation. Primer sequences for the multiplex PCR was partially digested to ligate barcode adapters (Ion P1 Adaptor and Ion XpressTM Bacode X, Life Technologies, Carlsbad, CA) followed by a bead-based nucleic acid purification system (AMPure® XP Reagent, Life Technologies, Carlsbad, CA). After confirmation that the final library fragment size peaked at 130 bp, the library fragments were clonally amplified by emulsion PCR (Ion PGM Template OT2, Life Technologies, Carlsbad, CA). The emulsion particles containing clonally amplified PCR fragments were then applied onto a semiconductor sequencing chip (Ion 316 Chip, Life Technologies, Carlsbad, CA) for massive parallel sequencing on an Ion PGM sequencer (Life Technologies, Carlsbad, CA).

### Target deep sequencing

The sequencing data were saved in BAM format for downstream analysis. The sequencing alignment was assessed with Torrent Suite V.3.6.2 Software (Life Technologies, Carlsbad, CA) to parse barcoded reads and align the reads to the reference genome (human genome build19; hg19). For the detection of variations in the targeted sequence, the extent of coverage of each amplicon was set to obtain at least the mean depth of 1400 x for primary tumors and 700 x for plasma DNA, where the Ion Torrent Variant Caller v3.6 was set at an allele frequency above 0.1% for a variant. An Integrative Genomics Viewer (IGV, https://www.broadinstitute.org/igv/) was also used to visualize the alignment, which allowed for us to inspect falsely defined variations by strand bias and sequencing errors.

### Identification and detection of genes for potential MMs

MMs from the primary tumors were designated to prioritize single nucleotide variants (SNVs) that were likely to be detected in ctDNA. The targeted sequencing from the Cancer Panel identified tumor-unique SNVs (i.e., somatic mutations) by comparing sequencing results of the primary tumor and corresponding PBMCs (i.e., germline polymorphisms). Briefly, the algorithm for identification of tumor-unique mutations is as follows: (a) Filter short reads (< 50 nt) using fastaq file for DNA from the tumor, PBMCs, and plasma; (b) Map filtered fragments on hg19 using Burrows-Wheeler Aligner for DNA from the tumor, PBMCs, and plasma; (c) Detect SNVs using GATK Unified Genotyper for DNA from the tumor or PBMCs; (d) List tumor-unique SNVs by comparing SNVs from the tumor and PBMCs; and (e) Identify tumor-unique mutations from the tumor-unique SNVs that were mapped on the target sequence from CHPv2. The entire process of algorithm execution takes six hours using an ordinary desktop computer (Intel Core 2 Duo Processors with 3 GB random accessing memory) for 1.5 GB of sequencing data. The resulting tumor-unique mutations may be used as ctDNA markers. Our in-house algorithm identifies primary tumor SNV fragments that are differentially detected from PBMC DNA. It allows for the selection of the fragments with high allele frequency, which holds a high likelihood of detection in ctDNA [11]. Of the resulting tumor-unique mutations at any variant frequencies of SNVs, MMs for each tumor were prioritized based on the following criteria: (a) more than 10 variant coverage; (b) more than 5 variant coverage if no mutations had more than 10 variant coverage; and (c) availability of validated QX200^TM^ Droplet Digital^TM^ PCR System (ddPCR, Bio-Rad Laboratories, Hercules, CA) primer and probe sequences (S1 Table). The allele frequency of MMs in plasma was monitored by ddPCR using the specific primer and probe sets.

### ddPCR

Each mixture was prepared with 20 μL reaction buffer, 2 x ddPCP SuperMix for Probes (Bio-Rad Laboratories, Hercules, CA), and 10 ng template DNA. The PCR reaction mixtures were separated into uniformly-sized emulsion droplets. The droplets were distributed into a 96-well microplate for use with a conventional thermal cycler. A standard PCR reaction was used as follows: 40 cycles of 94°C for 30 s and 55°C for 60 s; and a final extension at 98°C for 10 min, of which the annealing temperature was subject to change depending on the primers. The product was stored at 4°C. The PCR product was then placed into the QX200 droplet reader (Bio-Rad Laboratories, Hercules, CA) and the results were analyzed using QuantaSoft v1.6 (Bio-Rad Laboratories, Hercules, CA).

### Statistical analysis

Either JMP 10.0 (SAS Institute, Cary, NC) or Prism 6 (GraphPad Software Inc, La Jolla, CA) was used for statistical analysis. Clinicopathological and sequencing values and frequencies were analyzed using the χ^2^ test, Fisher’s exact test, and student *t*-test, depending on the subject groups.

## Results

### Patients

**Fig 1:**
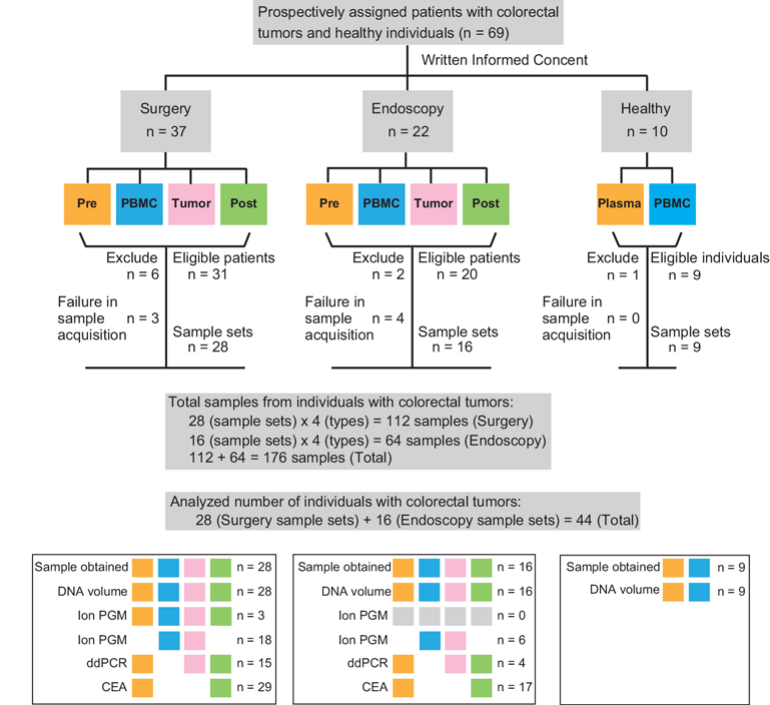
Sample collection diagram. All samples were collected prospectively. Samples were collected from the following three groups; Surgery, Endoscopy, and Healthy Volunteers. Surgery and Endoscopy groups contain Pre (pre-operative plasma), PBMCs, Tumor, and Post (post-operative plasma) samples. Samples from patients showing Stage IV disease, insufficient sample size, or insufficient extracted DNA amount were excluded from the study (detailed information in text). The color of each box indicates which procedures were used for analysis each type of sample.

Between May 2013 and August 2014, 37 patients with advanced colorectal cancer and 22 endoscopically-resectable colorectal tumors were consented for the study before a final histopathological diagnosis. The enrollment of patients/healthy individuals and overview of the study are presented (Fig 1). In the surgery group, six patients were ineligible: five patients were found to have Stage IV disease during surgery and one patient had multiple primary cancer lesions. Among eligible patients, the specimen acquisition process failed in three cases. Therefore, 28 full-sample sets were obtained from 31 eligible patients. In the endoscopy group, one patient refused to participate in the study, and one patient had renal failure after admission. Among eligible patients, four patients had tumors that were too small for sampling. Therefore, 16 full-sample sets were obtained from 20 eligible patients. Blood from 10 healthy individuals (i.e., patients between the age of 22 and 68 years; three females and seven males) was also collected using the same written informed consent. One volunteer was found to be pregnant after taking a blood sample and thereby ruled out. Overall, at least one type of sample were obtained from 60 individuals and a total of 176 samples of the set of four materials from 44 patients were obtained (Fig 1). Clinicopathological characteristics of patients (Table 1) and tumor marker levels (carcinoembryonic antigen; CEA) are summarized (S1 Fig). In the surgery group, 30 out of 31 (96.8%) eligible patients were observed for at least one year. Four out of the 30 (13.3%) patients relapsed and no patients died during the observation period (median: 14.3 months). No patients in endoscopy group had not yet visited our hospital after tumor resection as of February 2015.

**Table 1:** Clinicopathologic characteristics of eligible patients; Surgery & Endoscopic groups.

	Surgery Group	Endoscopy Group	
Factors	n = 31	n = 20	*P* value^c^
Gender			
Male	16	14	0.1927
Female	15	6	
Age			
65 ≤	17	12	0.7163
65 >	14	8	
Tumor site			
Colon	12	15	0.0112
Rectum	19	5	
Tumor size			
20mm ≤	30	8	0.0185
20mm >	1	12	
Histology			
tub^a^	28	6	<0.0001
muc^a^	1	0	
pap^a^	1	0	
por^a^	1	0	
tubular adenoma	-	14	
T factor^b^			
T0	0	14	<0.0001
Tis	0	4	
T1	1	2	
T2	9	0	
T3	20	0	
T4	1	0	
N factor^b^			
N0	14	0	NA^a^
N1	14	0	
N2	3	0	
NA	0	20	
M factor^b^			
M0	31	0	NA^a^
M1	0	0	
MA	0	20	
pStage^b^			
Tis	0	18	<0.0001
I	8	2	
II	6	0	
III	17	0	
IV	0	0	

^a^ Abbreviations: tub, tubular adenocarcinoma; muc, mucinous adenocarcinoma; pap, papillary adenocarcinoma; por, poorly differentiated adenocarcinoma; pStage, pathological stage; NA, Not Applicable.

^b^ TNM Classification of Malignant Tumors, 7th Edition.

^c^ *P* value was calculated by chi-square test.

### Quality assessment of extracted DNA

The median (range) total amount of DNA from primary tumors, plasma DNA, and PBMCs was 9.4 μg (1.4–12.0), 58.1 ng (15.4–915), and 4.7 μg (3.2–10.3), respectively. The plasma DNA yield was sufficient to perform downstream assays, including sequence library construction and ddPCR. The quantity of plasma DNA (range; 83–5,435 ng/ml per plasma) exhibited very high positive correlation (r = 0.9651, p < 0.0001) with the copy number of *LINE-1* (range; 3,050,985–232,689,225 copies/ml per plasma) (S2 Fig). Overall, our results show that 22.9 ng of DNA on average can be obtained from 1 ml plasma.

### Quality assessment of the Ion PGM sequencer

**Fig 2:**
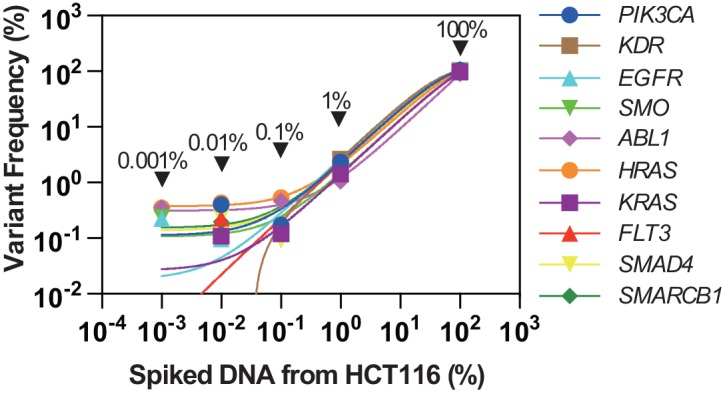
Sensitivity estimation of the Ion PGM. The horizontal axis indicates the concentration of spiked DNA from the HCT116 colon cancer cell line in the solution of DNA from a healthy non-cancer donor. HCT116 is known to possess several gene mutations and thus the concentration of the mutation fragment was serially diluted. The vertical axis is the allele frequency that is actually detected with the Ion PGM. Each color point indicates the detected allele frequency of cancer-associated mutations at the corresponding DNA concentration derived from HCT116 ranging from 0.001 to 100%. The names of the mutated genes are indicated in the legend on the right.

Prior to sequencing patient material, the sequencing quality of Ion PGM was assured by using serially diluted genomic DNA from the HCT116 human colon cancer cell line spiked into the solution of genomic DNA from PBMCs of a healthy volunteer (Fig 2). We first confirmed that HCT116 cells bear 10 gene mutations from the 50 genes of CHPv2 (S2 Table), while the healthy human volunteer DNA did not possess significant mutations. Based on publicly available information, 1177 mutations in HCT116 have been reported (https://cansar.icr.ac.uk/cansar/cell-lines/HCT-116/mutations/#). Among the 10 mutated genes found in the present study, 4 have been registered in the COSMIC database of HCT116, while the remaining 6 were novel. Notably, no known mutations were missed in the 50 genes covered by the primer sets in the CHPv2. To address the sensitivity, genomic DNA obtained from a healthy volunteer was spiked with genomic DNA from the HCT116 colon cancer cell line at four different concentrations (100, 1, 0.1, 0.01, and 0.001% in v/v) (Fig 2). The average sequence coverage of all amplicons for the listed concentrations was 1287.7 (100%), 1456.7 (1%), 1412.5 (0.1%), 1708.2 (0.01%), and 1464.3 (0.001%), respectively. In addition, coefficients of variations (CV) of variant frequencies of the mutated fragments were 33.4% (100%), 49.9% (1.0%), 125.6% (0.1%), 84.7% (0.01%), and 115.6% (0.001%), respectively. Overall, the reasonable linear range between the set concentrations and detected allele frequency with the Ion PGM sequencer appeared to be between 0.1 to 100%. Therefore, the sensitivity of the sequencing process for the variation frequencies using the Ion PGM is greater than 0.1% with sufficient sequence reads.

### Mutational spectrum of colorectal tumors identified by CHPv2

**Fig 3:**
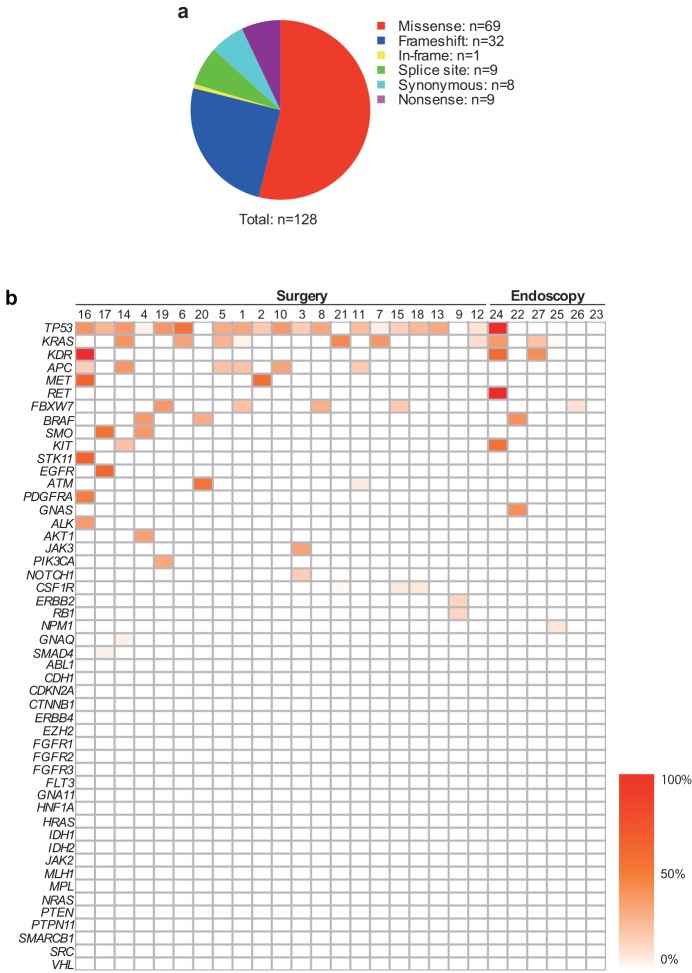
Mutation characteristics of colorectal tumors. a, Mutation types. Six types of mutations were detected with CHPv2. b, Tumor-unique mutation profile according to allele frequency. Each column denotes a tumor-unique mutation of an individual tumor. Each row denotes cancer-associated genes in CHPv2. The color indicates the variant allele frequency indicated in the color bar.

A total of 15,354,178 reads and 1,636,525,575 base sequence data were obtained from 27 primary tumors and corresponding PBMCs using an Ion PGM sequencer. The tumor-unique mutated genes were then identified using our in-house developed algorithm (see patients and methods). First we set the variant allele frequency > 0.1% and found that 440 of 885 gene alterations were tumor-unique mutations based on the comparison between PBMCs and primary tumors. Sequencing results of primary tumors obtained from the IonPGM were confirmed by ddPCR for samples that could be assessed (S3 Fig). For a stringent analysis, variant coverage is one of the important factors for data reliability (S4 Fig). Hence, analysis was performed with genes whose variant coverage was >10, resulting in a total of 128 gene point mutations (Fig 3A). Since some cases possessed multiple alterations in a single gene, the total number of altered genes in this study for analysis was 73. Therefore, the average mutation per tumor was 2.7 out of the 50 genes (mean ± 2 Standard Deviations: 2.7 ± 2.9). Twenty-six genes were mutated in at least 1 sample (26/50, 52%) while 14 genes (14/50, 28%) were mutated in only 1 sample, respectively. Frequently mutated genes included *TP53* (19/27, 70%), *KRAS* (10/27, 37%), and *APC* (6/27, 22%). Three cancer samples included all of these mutations, suggesting that alterations of genetic accumulation typical for an adenoma-carcinoma sequence may have occurred in these samples [16, 17] (Fig 3B). These observations seem to support previous reports from exome sequencing of colorectal tumors in terms of capturing mutational characteristics of colorectal tumors [18], suggesting that the 50 cancer-associated gene set reasonably recapitulates the mutational spectrum of the tumors. The mutation rate based on the multiplex PCR length obtained from CHPv2 and the number of mutations with coverage >10 (73 mutated genes) was approximately 2,246 per 10^6^ nucleotides (i.e., 207 primer pairs of average PCR product length 157bp), suggesting that the CHPv2 was enriched compared to the mutation detection rate from an exome-sequence, in which a majority of colorectal tumors showed 1–100 mutations per 10^6^ nucleotides [18].

### Detection of MMs in plasma DNA

**Fig 4:**
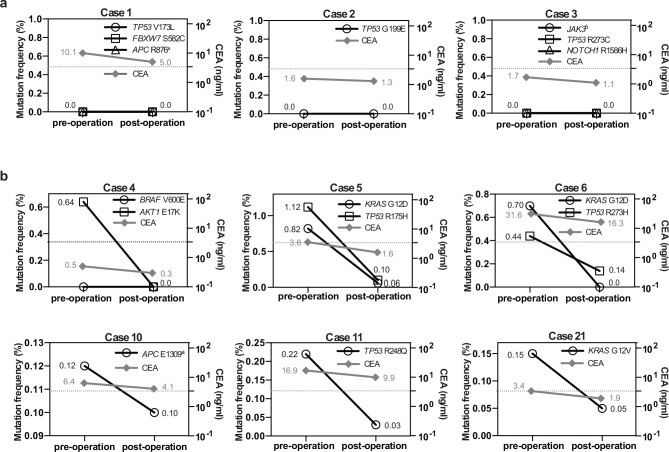
Dynamics of MMs and CEA in pre- and post-operation. a, MMs monitored with the mutation allele frequency using an Ion PGM. Three, one, and three MMs were used for monitoring cases 1, 2, and 3, respectively. The corresponding serum levels of CEA are shown. b, MMs monitored with the mutation allele frequency by ddPCR. One or two MMs were used for monitoring in the represented cases. The horizontal dotted line shows the upper limit of the normal range of CEA serum levels (3.4 ng/ml). Each number adjacent to each data point is the allele frequency for genes; and serum values for CEA. ^a^Stop codon, ^b^Splice site.

The median (range) plasma DNA levels of healthy individuals, endoscopically-resectable tumors, and advanced cancers were 4.2 (2.6–10.4), 6.8 (2.1–100.6), and 9.2 (3.8–228.8) ng/ml in plasma, respectively (S5 Fig). For mutation detection in plasma DNA, 66 MMs were selected from the 320 tumor-unique SNVs according to the criteria described in Patients and Methods. The following mutations, which have been reported in many different cancer types, appeared more than once in multiple tumors: *KRAS* (G12C) x2; *KRAS* (G12D) x4; *KRAS* (G12V) x3; *TP53* (R273C) x2; and *BRAF* (V600E) x3, resulting in a total of 57 unique MMs (S3 Table). MMs were first investigated using the Ion PGM for Cases 1, 2, and 3, but none of the eight MMs in plasma DNA showed a high enough variant coverage (Fig 4A and S4 Table). Although some genes demonstrated decreased allele frequency in a tumor burden-dependent manner, the extent of coverage was not reliably high enough in the present cases (S6A Fig).

Since the Ion PGM did not seem to be sensitive enough for detecting rare alleles, we decided to use ddPCR to detect MMs in pre- and post-operative plasma DNA. Although digital PCR requires a specific primer/probe set for each mutation followed by quality validation by qPCR [10], the digital PCR is at least 3 times more sensitive than that of deep sequencers [19]. In the present study, we were able to validate 19 unique primer/probe sets by qPCR for use in quantifying mutations in plasma by ddPCR (S1 Table). Since some cases had multiple MMs, the 19 validated ddPCR primer/probe sets represented a total of 24 MMs for 19 cases (S5 Table). Eleven mutations (in 9 patients) that matched primary tumors were apparently present (minimum allele frequency 0.032%) in pre-operational plasma (Fig 4B, S5 Table, and S6B Fig). In post-operative plasma DNA, 8 of 24 (33.3%) MMs demonstrated a decreasing trend that corresponded to 6 of 19 patients (31.6%), including two patients with multiple MMs (Fig 4B and S6B Fig). Importantly, 100% (8 of 8) of MMs with > 0.1% allele frequency in pre-operational plasma DNA exhibited a decrease in post-operation samples (Fig 4B), whereas none of the 16 MMs with less than 0.1% allele frequency in pre-operational plasma DNA exhibited a decrease in post-operative plasma DNA (Fig 4B and S6B Fig). The decreased trend obtained by MMs with > 0.1% allele frequency correlated well with serum CEA levels. There were 2 patients who had relapsed within the one year observation period (Case 5 and 6). Both cases exhibited a clear decrease of MMs in post-operative ctDNA (Fig 4B), but no remarkable mutational profile was identified in either case.

## Discussion

The set of gene mutations in a tumor is highly diverse. Therefore, an individualized set of tumor-derived mutated genes should be appropriate biomarkers for individual subjects. Whole genome analysis and exome sequence may not be cost-effective for this purpose, since more than 99.99% of the genome or exome sequence in primary tumors does not exhibit mutations [4, 18]. Here, we identified tumor-unique mutations with a CHPv2 on Ion PGM and subsequently monitored tumor burden using MMs with ddPCR starting from an extremely small amount of plasma DNA. Since our approach seems to be sufficient to obtain good quality mutational information compared to the whole genome or exome sequence technologies, it may be immediately applicable in clinical practice.

The utility of mutation detection in ctDNA has been reported in highly advanced colorectal cancer patients, the majority of whom experienced recurrence, progression, or death within one year after initial treatment [1, 2, 5, 9, 20, 21]. These highly advanced tumors (i.e., Stage IV) are considered to have a high risk of recurrence or progression [22], so the role of additional markers may be limited in current practice. In fact, the majority of colorectal cancer patients can be treated with curative intent (i.e., Stage II-III), whose 5-year disease-free rates have been reported to be roughly 70% [23, 24], suggesting that roughly 30% of the patients still require careful monitoring for relapse. Currently, CEA is one of the only molecular markers for routine use in monitoring post-operative follow-up [25]. However, it has been reported that the survival advantage by CEA monitoring and subsequent surgical treatment is likely to be small [26]. This observation is probably due to the fact that increased CEA levels are: (i) a poor predictor for local recurrence; and (ii) a relatively late event [27]. In contrast to CEA, ctDNA responds promptly, is specific to tumor burden, and is detectable regardless of histological type [2]. However, it should be noted that one of the important issues of using ctDNA in Stage II-III patients is the detection sensitivity. The prevalence of primary tumor-driven mutations in ctDNA has only a 0.1–10.0% variant allele frequency, even in highly advanced tumors [10, 11, 28]. Therefore, for ctDNA to be used as a biomarker for Stage II-III or even Stage I colorectal cancer patients, ideally the sensitivity is lower than 0.1% [19]. Recent advancements in digital genomic sequencing technologies, including beads, emulsion, amplification, and magnetics (BEAMing) [29], tagged-amplicon deep sequencing (Tam-Seq) [10], safe-sequencing system (Safe-SeqS) [30], error-suppressed multiplexed deep sequencing [31], and Duplex Sequencing [32] have approached this sensitivity demand. These methods are in fact highly accurate, but have not been fully applicable to search mutations with multiple amplicons from a limited copy number of templates such as ctDNA [30]. In the present study, we first identified tumor-unique mutations by Ion PGM, and subsequently these mutations were analyzed using ddPCR. The ddPCR requires primer/probe design and validation for previous identification of every mutation in the primary tumor but it does not require pool or deep sequencing. We confirmed that ddPCR was suitable for the quantitative measurement of rare variants at a mutant allele fraction of 0.1% or more (one mutant molecule in a background of 1000 wild-type molecules) [1, 33, 34]. For the practical use of ctDNA as a tumor burden monitoring marker, only a small number of certainly identified mutations from primary tumors could be reliable markers. Our current strategy is therefore reasonable for clinical tumor burden monitoring particularly for post-operative patients with curative intent.

Gene alterations involved in the early stages of tumorigenesis are apparently advantageous as MMs because they should be involved in the establishment of tumorigenic clones [4]. In principle, genetic heterogeneity of a tumor has been considered to be the result of heterogeneous accumulation of genetic alterations on the top of precancerous or early cancer lesions [35, 36]. In fact, mutations of *TP53*, *KRAS*, *KIT*, and *CDKN2A* were detected in endoscope group tumors as well as advanced cancers, suggesting that these mutations are carried over in the process of cancer development and spread out in the entire tumor mass. If a given mutation is associated with early cancer development of the tumor, then the mutation detection bias in ctDNA due to tumor heterogeneity should be minimized. However, the identification of genes that are specifically involved in the early development of individual tumors may be challenging. In the present study, it may be difficult to address clonal heterogeneity of a tumor in the mutation profiling with the small cancer-associated gene sequencing panel from a single biopsy per primary tumor. Ideally, all mutations, including those with low allele frequencies in the primary tumor from a single biopsy, should be examined in ctDNA. However, detection of extremely low allele frequency may not be feasible as yet due to the lack of ddPCR primer/probe sets for each single nucleotide change of all coding regions. Mutational profiling with multiple biopsies from a tumor may be an option to compensate for clonal heterogeneity, but this approach is as yet not possible for small tumors, such as polyps and resectable tumors. Therefore, assessment of clonal heterogeneity of a tumor may not be fully feasible in early cancers. In the meantime, mutations with high prevalence in primary tumors–the MMs from a cancer-associated gene sequencing panel in the present study–may be one of the best surrogates for this approach [11].

In summary, although deep sequencing is not currently feasible in daily practice and the existing primer-probe sets for ddPCR are far from complete, our strategy suggests that MMs in ctDNA seem to be a promising new class of individualized cancer biomarkers that can be detected to assess tumor burden in the context of surgical intervention.

## Supporting Information

S1 Fig Monitoring of serum concentration of CEA. Comparison of serum CEA levels taken pre- and post-surgery. The horizontal dotted line shows the upper limit of the standard range (3.4 ng/ml). The *p* value is calculated based on the student’s *t*-test.
(EPS)

S2 Fig A correlation between the concentration of plasma DNA and the copy number of *LINE-1* in plasma. Horizontal axis and vertical axis indicate plasma DNA concentration (ng/ml) and *LINE-1* copy number in plasma, respectively. Sample DNA from 12 cancer patients, 7 adenoma patients, and 4 healthy individuals. N/D, Not Detected.
(EPS)

S3 Fig Validation of the Ion PGM sequencing results by ddPCR. The horizontal axis indicates tumor mutation allele frequencies from CHPv2 in the Ion PGM. Detected mutations were validated by ddPCR with the corresponding mutant and wild-type probe sets (vertical axis). Each number indicates the MM ID in S3 Table. Correlation coefficient and corresponding *p* value are indicated.
(EPS)

S4 Fig Relationship between the extent of variant allele coverage and corresponding variant allele frequency. The horizontal axis indicates the log-scale variant coverage. The vertical axis indicates the corresponding variant allele frequency.
(EPS)

S5 Fig Concentrations of pre-operative plasma DNA from each group. The concentration of plasma DNA from healthy volunteers, patients from the endoscopy group, and patients from the surgery group. To examine the tumor burden, the concentration of pre-operative plasma DNA was compared. A student’s *t*-test was conducted for comparison. NS, Not Significant.
(EPS)

S6 Fig Dynamics of mutations and CEA levels in pre- and post-surgical samples. a, Tumor-unique mutations (including MMs) monitored by Ion PGM and CEA. b, MMs monitored by ddPCR and CEA. Note that all pre-operational mutation allele frequencies were below 0.1%. The horizontal dotted line shows the upper limit of the normal range of CEA serum levels (3.4 ng/ml). ^a^Stop codon, ^b^Synonymous, ^c^Splice site.
(EPS)

S1 Table Probe sets for ddPCR. (DOCX)

S2 Table Genes mutated in HCT116 cell line. (DOCX)

S3 Table Marker mutations in 27 primary tumor cases. (DOCX)

S4 Table Tumor-unique mutations in 3 cases by Ion PGM. (DOCX)

S5 Table Tumor and ctDNA concentrations of validated marker mutations in 19 cases by ddPCR. (DOCX)

## References

[1] Dawson SJ, Tsui DW, Murtaza M, Biggs H, Rueda OM, Chin SF, et al Analysis of circulating tumor DNA to monitor metastatic breast cancer. N Engl J Med. 2013;368(13):1199–209. 10.1056/NEJMoa1213261 23484797

[2] Diehl F, Schmidt K, Choti MA, Romans K, Goodman S, Li M, et al Circulating mutant DNA to assess tumor dynamics. Nat Med. 2008;14(9):985–90. 10.1038/nm.1789 18670422 PMC2820391

[3] Tomasetti C, Marchionni L, Nowak MA, Parmigiani G, Vogelstein B. Only three driver gene mutations are required for the development of lung and colorectal cancers. Proc Natl Acad Sci U S A. 2014. 10.1073/pnas.1421839112 PMC4291633 25535351

[4] Vogelstein B, Papadopoulos N, Velculescu VE, Zhou S, Diaz LA Jr, Kinzler KW. Cancer genome landscapes. Science. 2013;339(6127):1546–58. 10.1126/science.1235122 23539594 PMC3749880

[5] Diaz LA Jr, Williams RT, Wu J, Kinde I, Hecht JR, Berlin J, et al The molecular evolution of acquired resistance to targeted EGFR blockade in colorectal cancers. Nature. 2012;486(7404):537–40. 10.1038/nature11219 22722843 PMC3436069

[6] Gevensleben H, Garcia-Murillas I, Graeser MK, Schiavon G, Osin P, Parton M, et al Noninvasive detection of HER2 amplification with plasma DNA digital PCR. Clin Cancer Res. 2013;19(12):3276–84. 10.1158/1078-0432.CCR-12-3768 23637122 PMC6485473

[7] Oxnard GR, Paweletz CP, Kuang Y, Mach SL, O'Connell A, Messineo MM, et al Noninvasive detection of response and resistance in EGFR-mutant lung cancer using quantitative next-generation genotyping of cell-free plasma DNA. Clin Cancer Res. 2014;20(6):1698–705. 10.1158/1078-0432.CCR-13-2482 24429876 PMC3959249

[8] Spindler KL, Pallisgaard N, Andersen RF, Jakobsen A. Changes in mutational status during third-line treatment for metastatic colorectal cancer—results of consecutive measurement of cell free DNA, KRAS and BRAF in the plasma. Int J Cancer. 2014;135(9):2215–22. 10.1002/ijc.28863 24659028

[9] Spindler KL, Pallisgaard N, Vogelius I, Jakobsen A. Quantitative cell-free DNA, KRAS, and BRAF mutations in plasma from patients with metastatic colorectal cancer during treatment with cetuximab and irinotecan. Clin Cancer Res. 2012;18(4):1177–85. 10.1158/1078-0432.CCR-11-0564 22228631

[10] Forshew T, Murtaza M, Parkinson C, Gale D, Tsui DW, Kaper F, et al Noninvasive identification and monitoring of cancer mutations by targeted deep sequencing of plasma DNA. Sci Transl Med. 2012;4(136):136ra68 10.1126/scitranslmed.3003726 22649089

[11] Murtaza M, Dawson SJ, Tsui DW, Gale D, Forshew T, Piskorz AM, et al Non-invasive analysis of acquired resistance to cancer therapy by sequencing of plasma DNA. Nature. 2013;497(7447):108–12. 10.1038/nature12065 23563269

[12] Elbeik T, Nassos P, Kipnis P, Haller B, Ng VL. Evaluation of the VACUTAINER PPT Plasma Preparation Tube for use with the Bayer VERSANT assay for quantification of human immunodeficiency virus type 1 RNA. J Clin Microbiol. 2005;43(8):3769–71. 16081908 10.1128/JCM.43.8.3769-3771.2005 PMC1233973

[13] Salimnia H, Moore EC, Crane LR, Macarthur RD, Fairfax MR. Discordance between viral loads determined by Roche COBAS AMPLICOR human immunodeficiency virus type 1 monitor (version 1.5) Standard and ultrasensitive assays caused by freezing patient plasma in centrifuged becton-dickinson vacutainer brand plasma preparation tubes. J Clin Microbiol. 2005;43(9):4635–9. 16145119 10.1128/JCM.43.9.4635-4639.2005 PMC1234093

[14] Yu J, Miller R, Zhang W, Sharma M, Holtschlag V, Watson MA, et al Copy-number analysis of topoisomerase and thymidylate synthase genes in frozen and FFPE DNAs of colorectal cancers. Pharmacogenomics. 2008;9(10):1459–66. 10.2217/14622416.9.10.1459 18855534 PMC2575840

[15] Singh RR, Patel KP, Routbort MJ, Reddy NG, Barkoh BA, Handal B, et al Clinical validation of a next-generation sequencing screen for mutational hotspots in 46 cancer-related genes. J Mol Diagn. 2013;15(5):607–22. 10.1016/j.jmoldx.2013.05.003 23810757

[16] Dickinson BT, Kisiel J, Ahlquist DA, Grady WM. Molecular markers for colorectal cancer screening. Gut. 2015;64(9):1485–94. 10.1136/gutjnl-2014-308075 25994221 PMC4765995

[17] Fearon ER, Hamilton SR, Vogelstein B. Clonal analysis of human colorectal tumors. Science. 1987;238(4824):193–7. 2889267 10.1126/science.2889267

[18] Cancer Genome Atlas N. Comprehensive molecular characterization of human colon and rectal cancer. Nature. 2012;487(7407):330–7. 10.1038/nature11252 22810696 PMC3401966

[19] Diaz LA Jr, Bardelli A. Liquid biopsies: genotyping circulating tumor DNA. J Clin Oncol. 2014;32(6):579–86. 10.1200/JCO.2012.45.2011 24449238 PMC4820760

[20] Perkins G, Yap TA, Pope L, Cassidy AM, Dukes JP, Riisnaes R, et al Multi-purpose utility of circulating plasma DNA testing in patients with advanced cancers. PLoS One. 2012;7(11):e47020 10.1371/journal.pone.0047020 23144797 PMC3492590

[21] Spindler KL, Appelt AL, Pallisgaard N, Andersen RF, Brandslund I, Jakobsen A. Cell-free DNA in healthy individuals, noncancerous disease and strong prognostic value in colorectal cancer. Int J Cancer. 2014;135(12):2984–91. 10.1002/ijc.28946 24798213

[22] Brenner H, Kloor M, Pox CP. Colorectal cancer. The Lancet. 2014;383(9927):1490–502. 10.1016/S0140-6736(13)61649-9 24225001

[23] Andre T, Boni C, Navarro M, Tabernero J, Hickish T, Topham C, et al Improved overall survival with oxaliplatin, fluorouracil, and leucovorin as adjuvant treatment in stage II or III colon cancer in the MOSAIC trial. J Clin Oncol. 2009;27(19):3109–16. 10.1200/JCO.2008.20.6771 19451431

[24] Yothers G, O'Connell MJ, Allegra CJ, Kuebler JP, Colangelo LH, Petrelli NJ, et al Oxaliplatin as adjuvant therapy for colon cancer: updated results of NSABP C-07 trial, including survival and subset analyses. J Clin Oncol. 2011;29(28):3768–74. 10.1200/JCO.2011.36.4539 21859995 PMC3188282

[25] Mutch MG. Molecular profiling and risk stratification of adenocarcinoma of the colon. J Surg Oncol. 2007;96(8):693–703. 18081153 10.1002/jso.20915

[26] Primrose JN, Perera R, Gray A, Rose P, Fuller A, Corkhill A, et al Effect of 3 to 5 years of scheduled CEA and CT follow-up to detect recurrence of colorectal cancer: the FACS randomized clinical trial. JAMA. 2014;311(3):263–70. 10.1001/jama.2013.285718 24430319

[27] McArdle C. ABC of colorectal cancer: primary treatment-does the surgeon matter? BMJ. 2000;321(7269):1121–3. 11061734 10.1136/bmj.321.7269.1121 PMC1118899

[28] Diehl F, Li M, Dressman D, He Y, Shen D, Szabo S, et al Detection and quantification of mutations in the plasma of patients with colorectal tumors. Proc Natl Acad Sci U S A. 2005;102(45):16368–73. 16258065 10.1073/pnas.0507904102 PMC1283450

[29] Dressman D, Yan H, Traverso G, Kinzler KW, Vogelstein B. Transforming single DNA molecules into fluorescent magnetic particles for detection and enumeration of genetic variations. Proc Natl Acad Sci U S A. 2003;100(15):8817–22. 12857956 10.1073/pnas.1133470100 PMC166396

[30] Kinde I, Wu J, Papadopoulos N, Kinzler KW, Vogelstein B. Detection and quantification of rare mutations with massively parallel sequencing. Proc Natl Acad Sci U S A. 2011;108(23):9530–5. 10.1073/pnas.1105422108 21586637 PMC3111315

[31] Narayan A, Carriero NJ, Gettinger SN, Kluytenaar J, Kozak KR, Yock TI, et al Ultrasensitive measurement of hotspot mutations in tumor DNA in blood using error-suppressed multiplexed deep sequencing. Cancer Res. 2012;72(14):3492–8. 10.1158/0008-5472.CAN-11-4037 22581825 PMC3426449

[32] Schmitt MW, Kennedy SR, Salk JJ, Fox EJ, Hiatt JB, Loeb LA. Detection of ultra-rare mutations by next-generation sequencing. Proc Natl Acad Sci U S A. 2012;109(36):14508–13. 10.1073/pnas.1208715109 22853953 PMC3437896

[33] Hindson BJ, Ness KD, Masquelier DA, Belgrader P, Heredia NJ, Makarewicz AJ, et al High-throughput droplet digital PCR system for absolute quantitation of DNA copy number. Anal Chem. 2011;83(22):8604–10. 10.1021/ac202028g 22035192 PMC3216358

[34] Yung TK, Chan KC, Mok TS, Tong J, To KF, Lo YM. Single-molecule detection of epidermal growth factor receptor mutations in plasma by microfluidics digital PCR in non-small cell lung cancer patients. Clin Cancer Res. 2009;15(6):2076–84. 10.1158/1078-0432.CCR-08-2622 19276259

[35] Jones S, Chen WD, Parmigiani G, Diehl F, Beerenwinkel N, Antal T, et al Comparative lesion sequencing provides insights into tumor evolution. Proc Natl Acad Sci U S A. 2008;105(11):4283–8. 10.1073/pnas.0712345105 18337506 PMC2393770

[36] Yachida S, Jones S, Bozic I, Antal T, Leary R, Fu B, et al Distant metastasis occurs late during the genetic evolution of pancreatic cancer. Nature. 2010;467(7319):1114–7. 10.1038/nature09515 20981102 PMC3148940

