# Supplementary material for: Individualized Mutation Detection in Circulating Tumor DNA for Monitoring Colorectal Tumor Burden Using a Cancer-Associated Gene Sequencing Panel
Source: PLoS One. 2016 Jan 4;11(1):e0146275. doi: 10.1371/journal.pone.0146275 (PMC4699643; doi:10.1371/journal.pone.0146275)
Supplement: S1 Table — (DOCX) [file pone.0146275.s007.docx]

**S1 Table.** Probe sets for ddPCR

| CASE | Gene | Position | COSMIC ID | Amino Acid Change | Validation by qPCR | Analyzed by ddPCR | WT probe (Cat.No.) | Mut probe (Cat.No.) |
| --- | --- | --- | --- | --- | --- | --- | --- | --- |
| 4 | *BRAF* | 140453136 | COSM476 | V600E | Yes | Yes | 186-3100^a^ | |
| 4 | *AKT1* | 105246551 | COSM33765 | E17K | Yes | Yes | dHsaCP2000032^b^ | dHsaCP200003^b^ |
| 5 | *TP53* | 7578406 | COSM10648 | R175H | Yes | Yes | dHsaCP2000106^b^ | dHsaCP2000105^b^ |
| 5 | *KRAS* | 25398284 | COSM521 | G12D | Yes | Yes | dHsaCP2000110^b^ | dHsaCP2000109^b^ |
| 6 | *TP53* | 7577120 | COSM10660 | R273H | Yes | Yes | dHsaCP2500539^b^ | dHsaCP2500538^b^ |
| 6 | *KRAS* | 25398284 | COSM521 | G12D | Yes | Yes | 186-3112^a^ | |
| 7 | *KRAS* | 25398285 | COSM516 | G12C | Yes | Yes | 186-3111^a^ | |
| 8 | *TP53* | 7577121 | COSM10659 | R273C | Yes | Yes | dHsaCP2500539^b^ | dHsaCP2500538^b^ |
| 9 | *ERBB2* | 37880220 | COSM14060 | L755S | Yes | Yes | dHsaIS2505135^b^ | dHsaIS2505134^b^ |
| 10 | *TP53* | 7577124 | COSM10859 | V272L | No | No | dHsaIS2504907^b^ | dHsaIS2504906^b^ |
| 10 | *APC* | 112175216 | COSM18775 | E1309 | Yes | Yes | dHsaIS2505253^b^ | dHsaIS2505252^b^ |
| 11 | *TP53* | 7577538 | COSM10662 | R248Q | Yes | Yes | dHsaCP2000128^b^ | dHsaCP2000127^b^ |
| 12 | *KRAS* | 25398281 | COSM532 | G13D | Yes | Yes | 186-3116^a^ | |
| 12 | *TP53* | 7578394 | COSM10889 | H179R | Yes | No | dHsaCP2000126 ^b^ | dHsaCP2000125 ^b^ |
| 13 | *TP53* | 7578268 | COSM44571 | L194R | Yes | Yes | dHsaIS2500687 ^b^ | dHsaIS2500686 ^b^ |
| 14 | *KRAS* | 25398284 | COSM520 | G12V | Yes | Yes | 186-3108^a^ | |
| 14 | *TP53* | 7578190 | COSM10758 | Y220C | Yes | No | dHsaCP2500537^b^ | dHsaCP2500536^b^ |
| 16 | *TP53* | 7577547 | COSM11196 | G245V | No | No | dHsaCP2500555^b^ | dHsaCP2500554^b^ |
| 17 | *TP53* | 7577568 | COSM11059 | C238Y | Yes | Yes | dHsaIS2500629^b^ | dHsaIS2500628^b^ |
| 18 | *TP53* | 7577547 | COSM11196 | G245V | No | No | dHsaCP2500556^b^ | dHsaCP2500554^b^ |
| 19 | *TP53* | 7578404 | COSM44146 | C176S | Yes | Yes | dHsaIS2501179^b^ | dHsaIS2501178^b^ |
| 19 | *PIK3CA* | 178936091 | COSM763 | E545K | Yes | Yes | dHsaCP2000076^b^ | dHsaCP2000075^b^ |
| 20 | *BRAF* | 140453136 | COSM476 | V600E | Yes | Yes | 186-3100^a^ | |
| 21 | *KRAS* | 25398284 | COSM520 | G12V | Yes | Yes | 186-3108^a^ | |
| 22 | *GNAS* | 57484421 | COSM27895 | R201H | Yes | Yes | dHsaIS2501325^b^ | dHsaIS2501324^b^ |
| 22 | *BRAF* | 140453136 | COSM476 | V600E | Yes | Yes | 186-3100^a^ | |
| 24 | *TP53* | 7578446 | COSM44413 | I162V | No | No | dHsaIS2502557^b^ | dHsaIS2502556^b^ |
| 24 | *KRAS* | 25398284 | COSM521 | G12D | Yes | Yes | 186-3112^a^ | |
| 25 | *BRAF* | 140453136 | COSM476 | V600E | Yes | Yes | 186-3100^a^ | |
| 27 | *KRAS* | 25398284 | COSM521 | G12D | Yes | Yes | 186-3112^a^ | |
| 27 | *KRAS* | 25398285 | COSM516 | G12C | Yes | No | 186-3111^a^ | |

Abbreviations: qPCR, quantitative PCR; ddPCR, doroplet digital PCR; WT, Wild-type; Mut, Mutation

^a^Catalogue number in Japan (Bio-Rad Laboratories)

^b^Catalogue number in U. S. A. (Bio-Rad Laboratories)
