# Supplementary material for: Individualized Mutation Detection in Circulating Tumor DNA for Monitoring Colorectal Tumor Burden Using a Cancer-Associated Gene Sequencing Panel
Source: PLoS One. 2016 Jan 4;11(1):e0146275. doi: 10.1371/journal.pone.0146275 (PMC4699643; doi:10.1371/journal.pone.0146275)
Supplement: S2 Table — (DOCX) [file pone.0146275.s008.docx]

**S2 Table** Genes mutated in HCT116 cell line

| Genes | Position | Nucleotide mutation | Amino Acid Change | COSMIC ID | Coverage | Variant Coverage | Variant frequency (%) | |
| --- | --- | --- | --- | --- | --- | --- | --- | --- |
| *ABL1* | 133738370 | A > G | Y257C | COSM1674905 | 1653 | 743 | 49.9 |  |
| *KRAS* | 25398281 | C > T | G13D | COSM532 | 1624 | 788 | 48.5 |  |
| *PIK3CA* | 178952085 | A > G | H1047R | COSM775 | 1065 | 555 | 52.1 |  |
| *SMO* | 128846374 | G > A | V404M | COSM13148 | 1340 | 641 | 47.8 |  |
| *EGFR*^a^ | 55249063 | G > A | Synonymous | COSM1451600 | 670 | 669 | 99.9 |  |
| *FLT3*^a^ | 28602367 | C > T | Synonymous | COSM2070142 | 1505 | 815 | 54.2 |  |
| *HRAS*^a^ | 534242 | A > G | Synonymous | COSM249860 | 815 | 815 | 100.0 |  |
| *KDR*^a^ | 55946354 | G > T | Splice site | - | 1467 | 754 | 51.4 |  |
| *SMAD4*^a^ | 48586344 | C > T | Splice site | - | 1966 | 948 | 48.2 |  |
| *SMARCB1*^a^ | 24176287 | G > A | Splice site | COSM1090 | 772 | 419 | 54.3 |  |

^a^Variations not previously reported
