# Supplementary material for: Individualized Mutation Detection in Circulating Tumor DNA for Monitoring Colorectal Tumor Burden Using a Cancer-Associated Gene Sequencing Panel
Source: PLoS One. 2016 Jan 4;11(1):e0146275. doi: 10.1371/journal.pone.0146275 (PMC4699643; doi:10.1371/journal.pone.0146275)
Supplement: S3 Table — (DOCX) [file pone.0146275.s009.docx]

**S3 Table.** Marker mutations in 27 primary tumor cases

| Case | TNM^a^ | Stage^b^ | MM ID | Gene | Position | Nucleotide mutation | Amino Acid Change | COSMIC ID | Cov | Var. Cov | Var.freq (%) | Mutated in other cancers^c^ |
| --- | --- | --- | --- | --- | --- | --- | --- | --- | --- | --- | --- | --- |
| 1 | T2N0M0 | I | 1 | *TP53* | 7578413 | G>T | V173L | COSM43559 | 1094 | 283 | 25.9 | breast, lung, esophagus, stomach, vulvar, other |
|  |  |  | 2 | *FBXW7* | 153245447 | A>G | S582C | - | 1685 | 285 | 16.9 | NA |
|  |  |  | 3 | *APC* | 112173917 | C>T | R876^c^ | COSM18852 | 1291 | 212 | 16.4 | breast, endometrium, lung, small intestine |
|  |  |  |  |  |  |  |  |  |  |  |  |  |
| 2 | T3N0M0 | IIA | 4 | *TP53* | 7578253 | C>T | G199E | COSM43989 | 739 | 104 | 14.1 | breast, lung, peritoneum, urinary tract |
|  |  |  |  |  |  |  |  |  |  |  |  |  |
| 3 | T3N2aM0 | IIIB | 5 | *JAK3* | 17945643 | C>T | Splice site | - | 664 | 180 | 27.1 | NA |
|  |  |  | 6 | *TP53* | 7577121 | G>A | R273C | COSM10659 | 553 | 73 | 13.2 | breast, liver, lung, ovary, pancreas, other |
|  |  |  | 7 | *NOTCH1* | 139399386 | C>T | R1586H | - | 587 | 76 | 12.9 | NA |
|  |  |  |  |  |  |  |  |  |  |  |  |  |
| 4 | T3N0M0 | IIA | 8 | *BRAF* | 140453136 | A>T | V600E | COSM476 | 890 | 276 | 31 | biliary tract, thyroid, upper aerodigestive tract, urinary tract |
|  |  |  | 9 | *SMO* | 128845165 | C>T | P220L | - | 1248 | 380 | 30.4 | NA |
|  |  |  | 10 | *AKT1* | 105246551 | C>T | E17K | COSM33765 | 915 | 254 | 27.8 | breast, endometrium, lung, thyroid, urinary tract, other |
|  |  |  |  |  |  |  |  |  |  |  |  |  |
| 5 | T3N1bM0 | IIIB | 11 | *TP53* | 7578406 | C>T | R175H | COSM10648 COSM99914 COSM99022 | 957 | 233 | 24.3 | breast, esophagus, ovary, pancreas, stomach, other |
|  |  |  | 12 | *KRAS* | 25398284 | C>T | G12D | COSM521 | 1122 | 241 | 21.5 | biliary tract, lung, ovary, pancreas, stomach, other |
|  |  |  | 13 | *APC* | 112173917 | C>T | R876^c^ | COSM18852 | 1156 | 193 | 16.7 | breast, endometrium, lung, small intestine |
|  |  |  |  |  |  |  |  |  |  |  |  |  |
| 6 | T3N1aM0 | IIIB | 14 | *TP53* | 7577120 | C>T | R273H | COSM10660 COSM99729 | 322 | 147 | 45.7 | breast, lung, esophagus, ovary, pancreas, other |
|  |  |  | 15 | *KRAS* | 25398284 | C>T | G12D | COSM521 | 971 | 259 | 26.7 | biliary tract, lung, ovary, pancreas, stomach, other |
|  |  |  |  |  |  |  |  |  |  |  |  |  |
| 7 | T3N0M0 | IIA | 16 | *KRAS* | 25398285 | C>A | G12C | COSM516 | 1750 | 563 | 32.2 | biliary tract, endometrium, lung, pancreas, urinary tract, other |
|  |  |  |  |  |  |  |  |  |  |  |  |  |
| 8 | T2N0M0 | I | 17 | *TP53* | 7577121 | G>A | R273C | COSM10659 COSM99933 | 591 | 151 | 25.5 | breast, liver, lung, ovary, pancreas, other |
|  |  |  | 18 | *FBXW7* | 153247366 | C>A | R479E | - | 1606 | 363 | 22.6 | NA |
|  |  |  |  |  |  |  |  |  |  |  |  |  |
| 9 | T3N1aM0 | IIIB | 19 | *ERBB2* | 37880220 | A>G | L755S | COSM14060 | 1849 | 201 | 10.9 | breast, endometrium, lung, small intestine, stomach, other |
|  |  |  | 20 | *RB1* | 48955573 | G>A | W563^b^ | - | 893 | 91 | 10.2 | NA |
|  |  |  |  |  |  |  |  |  |  |  |  |  |
| 10 | T2N1AM0 | IIIA | 21 | *TP53* | 7577124 | C>A | V272L | COSM10859 | 506 | 157 | 31 | breast, lung, esophagus, ovary, pancreas, other |
|  |  |  | 22 | *APC* | 112175216 | G>T | E1309^b^ | COSM18775 | 963 | 256 | 26.6 | None |
|  |  |  |  |  |  |  |  |  |  |  |  |  |
| 11 | T3N1bM0 | IIIB | 23 | *TP53* | 7577538 | C>T | R248Q | COSM99020 COSM99602 COSM10662 | 1070 | 202 | 18.9 | breast, esophagus, liver, lung, stomach, other |
|  |  |  | 24 | *APC* | 112173917 | C>T | R876^b^ | COSM18852 | 1161 | 158 | 13.6 | breast, endometrium, lung, small intestine |
|  |  |  |  |  |  |  |  |  |  |  |  |  |
| 12 | T3N1bM0 | IIIB | 25 | *KRAS* | 25398281 | C>T | G13D | COSM532 | 1417 | 112 | 7.9 | biliary tract, breast, pancreas, prostate, stomach, other |
|  |  |  | 26 | *TP53* | 7578394 | T>C | H179R | COSM10889 | 903 | 57 | 6.3 | breast, esophagus, lung, ovary, pancreas, other |
|  |  |  |  |  |  |  |  |  |  |  |  |  |
| 13 | T2N0M0 | I | 27 | *TP53* | 7578268 | A>C | L194R | COSM44571 | 950 | 236 | 24.8 | breast, liver, lung, esophagus, ovary, other |
|  |  |  |  |  |  |  |  |  |  |  |  |  |
| 14 | T3N1bM0 | IIIB | 28 | *KRAS* | 25398284 | G>T | G12V | COSM520 | 1180 | 389 | 33 | biliary tract, breast, lung, pancreas, stomach, other |
|  |  |  | 29 | *APC* | 112175339 | A>T | K1333^b^ | - | 1149 | 367 | 31.9 | NA |
|  |  |  | 30 | *TP53* | 7578190 | T>C | Y220C | COSM10758 | 608 | 183 | 30.1 | breast, liver, lung, esophagus, ovary, other |
|  |  |  | 31 | *KIT* | 55599358 | C>T | Synonymous | - | 943 | 163 | 17.3 | NA |
|  |  |  |  |  |  |  |  |  |  |  |  |  |
| 15 | T3N0M0 | IIA | 32 | *FBXW7* | 153247289 | G>C | R505G | COSM99604  COSM99606 | 1729 | 246 | 14.2 | cervix, esophagus, lung, upper aerodigestive tract, urinary tract, other |
|  |  |  | 33 | *TP53* | 7577085 | C>T | E285K | COSM10722  COSM137087 | 595 | 75 | 12.6 | breast, liver, lung, esophagus, urinary tract, other |
|  |  |  |  |  |  |  |  |  |  |  |  |  |
| 16 | T3N2aM0 | IIIB | 34 | *KDR* | 55972974 | T>A | Q472H | - | 1096 | 100 | 100 | NA |
|  |  |  | 35 | *SKT11* | 1220321 | T>C | Splice site | - | 381 | 209 | 54.9 | NA |
|  |  |  | 36 | *MET* | 116339672 | C>T | Synonymous | - | 1274 | 682 | 53.5 | NA |
|  |  |  | 37 | *MET* | 116340262 | A>G | N375S | - | 661 | 334 | 50.5 | NA |
|  |  |  | 38 | *PDGFRA* | 55152040 | C>T | V824V | COSM22413 | 1091 | 456 | 41.8 | skin |
|  |  |  | 39 | *TP53* | 7574003 | G>A | R342^b^ | COSM99721  COSM11073 | 522 | 172 | 33 | breast, liver, lung, esophagus, pancreas, other |
|  |  |  | 40 | *ALK* | 29443617 | C>G | Synonymous | - | 606 | 180 | 29.7 | NA |
|  |  |  | 41 | *APC* | 112175423 | C>T | Q1378^b^ | COSM18862 | 1001 | 126 | 12.6 | small intestine, stomach |
|  |  |  |  |  |  |  |  |  |  |  |  |  |
| 17 | T3N1bM0 | IIIB | 42 | *EGFR* | 55249063 | G>A | Synonymous | - | 516 | 270 | 52.3 | NA |
|  |  |  | 43 | *SMO* | 128845088 | A>G | Synonymous | - | 1083 | 506 | 46.7 | NA |
|  |  |  | 44 | *TP53* | 7577568 | C>T | C238Y | COSM11059 | 1603 | 325 | 20.3 | breast, lung, esophagus, ovary, other |
|  |  |  |  |  |  |  |  |  |  |  |  |  |
| 18 | T2N0M0 | I | 45 | *TP53* | 7577547 | C>A | G245V | COSM11196 | 1588 | 318 | 20.0 | breast, liver, lung, esophagus, upper aerodigestive tract, other |
|  |  |  |  |  |  |  |  |  |  |  |  |  |
| 19 | T2N0M0 | I | 46 | *FBXW7* | 153245453 | G>A | H580Y | - | 1978 | 621 | 31.4 | NA |
|  |  |  | 47 | *TP53* | 7578404 | A>T | C176S | COSM44146 | 1992 | 622 | 31.2 | breast, liver, lung, esophagus, pancreas, other |
|  |  |  | 48 | *PIK3CA* | 178936091 | G>A | E545K | COSM763 COSM125370 | 1099 | 278 | 25.3 | breast, lung, esophagus, stomach, upper aerodigestive tract, other |
|  |  |  |  |  |  |  |  |  |  |  |  |  |
| 20 | T3N1bM0 | IIIB | 49 | *ATM* | 108204684 | A>G | M2667V | - | 125 | 57 | 45.6 | NA |
|  |  |  | 50 | *BRAF* | 140453136 | A>T | V600E | COSM476 | 1484 | 355 | 23.9 | biliary tract, thyroid, upper aerodigestive tract, urinary tract, |
|  |  |  |  |  |  |  |  |  |  |  |  |  |
| 21 | T3N0M0 | IIA | 51 | *KRAS* | 25398284 | G>T | G12V | COSM520 | 1998 | 752 | 37.6 | biliary tract, breast, lung, pancreas, stomach, other |
|  |  |  |  |  |  |  |  |  |  |  |  |  |
| 22 | Adenoma | - | 52 | *GNAS* | 57484421 | G>A | R201H | COSM27895 | 423 | 146 | 34.5 | cervix, kidney, liver, pancreas, stomach, otehr |
|  |  |  | 53 | *BRAF* | 140453136 | A>T | V600E | COSM476 | 1077 | 364 | 33.8 | biliary tract, thyroid, upper aerodigestive tract, urinary tract, |
|  |  |  |  |  |  |  |  |  |  |  |  |  |
| 23 | Adenoma | - | 54 | *CSF1R* | 149433596 | T>G | Splice site | - | 326 | 8 | 2.5 | NA |
|  |  |  |  |  |  |  |  |  |  |  |  |  |
| 24 | Adenoma | - | 55 | *RET* | 43613843 | G>T | Synonymous | - | 1184 | 1184 | 100 | NA |
|  |  |  | 56 | *TP53* | 7579472 | G>C | P72R | - | 399 | 389 | 97.5 | NA |
|  |  |  | 57 | *TP53* | 7578446 | T>C | I162V | COSM44413 | 903 | 456 | 50.5 | ovary |
|  |  |  | 58 | *KDR* | 55946354 | G>T | Splice site | - | 1861 | 921 | 49.5 | NA |
|  |  |  | 59 | *KIT* | 55593464 | A>C | M541L | COSM28026 | 1996 | 936 | 46.9 | skin, stomach |
|  |  |  | 60 | *KRAS* | 25398284 | C>T | G12D | COSM521 | 1803 | 548 | 30.4 | biliary tract, lung, ovary, pancreas, stomach, other |
|  |  |  |  |  |  |  |  |  |  |  |  |  |
| 25 | T1NXMX | NA | 61 | *NPM1* | 170837602 | T>A | Splice site | - | 1099 | 63 | 5.7 | NA |
|  |  |  | 62 | *KRAS* | 25398284 | C>A | G12V | COSM520 | 1998 | 27 | 1.4 | biliary tract, breast, lung, pancreas, stomach, other |
|  |  |  |  |  |  |  |  |  |  |  |  |  |
| 26 | Adenoma | - | 63 | *FBXW7* | 153247366 | C>G | R479P | - | 1999 | 138 | 6.9 | NA |
|  |  |  |  |  |  |  |  |  |  |  |  |  |
| 27 | Adenoma | - | 64 | *KDR* | 55980239 | C>T | Splice site | - | 540 | 193 | 35.7 | NA |
|  |  |  | 65 | *KRAS* | 25398284 | C>T | G12D | COSM521 | 1667 | 276 | 16.6 | biliary tract, lung, ovary, pancreas, stomach, otehr |
|  |  |  | 66 | *KRAS* | 25398285 | C>A | G12C | COSM516 | 1430 | 226 | 15.8 | biliary tract, endometrium, lung, pancreas, urinary tract, other |

Abbreviations: NA, Not Applicable; MM ID, Mutation Maker ID; cov., coverage; var. cov., variant coverage.

^a^TNM Classification of Malignant Tumors, 7th Edition

^b^Stop codon

^c^Data obtained from COSMIC database (http://cancer.sanger.ac.uk/cosmic).
