# Supplementary material for: Individualized Mutation Detection in Circulating Tumor DNA for Monitoring Colorectal Tumor Burden Using a Cancer-Associated Gene Sequencing Panel
Source: PLoS One. 2016 Jan 4;11(1):e0146275. doi: 10.1371/journal.pone.0146275 (PMC4699643; doi:10.1371/journal.pone.0146275)
Supplement: S4 Table — (DOCX) [file pone.0146275.s010.docx]

**S4 Table.** Tumor-unique mutations in 3 cases by Ion PGM

| Case | TNM^a^ | pStage | Gene | Position | Nucleotide mutation | Amino Acid Change | COSMIC ID | Tumor | | | Pre-operation in cfDNA | | | Post-operation in cfDNA | | |
| --- | --- | --- | --- | --- | --- | --- | --- | --- | --- | --- | --- | --- | --- | --- | --- | --- |
|  |  |  |  |  |  |  |  | Cov | Var. Cov. | Var. freq. | Cov. | Var. Cov. | Var. freq. | Cov. | Var. Cov. | Var. freq. |
| 1 | T2N0M0 | I | *TP53* | 7578413 | G>T | V173L | COSM43559 | 1094 | 283 | 25.87 | 696 | 0 | 0 | 328 | 0 | 0 |
|  |  |  | *FBXW7* | 153245447 | A>G | S582C | - | 1685 | 285 | 16.91 | 0 | 0 | 0 | 0 | 0 | 0 |
|  |  |  | *APC* | 112173917 | C>T | R876^b^ | COSM18852 | 1291 | 212 | 16.42 | 863 | 0 | 0 | 437 | 0 | 0 |
|  |  |  | *TP53* | 7579473 | G>C | P72S | COSM44018 | 512 | 19 | 3.71 | 227 | 7 | 3.08 | 98 | 0 | 0 |
|  |  |  | *KRAS* | 25398285 | C>A | G12C | COSM516 | 1596 | 45 | 2.82 | 433 | 0 | 0 | 184 | 0 | 0 |
|  |  |  |  |  |  |  |  |  |  |  |  |  |  |  |  |  |
| 2 | T3N0M0 | IIA | *TP53* | 7578253 | C>T | G199E | COSM43989 | 739 | 104 | 14.07 | 1277 | 0 | 0 | 857 | 0 | 0 |
|  |  |  |  |  |  |  |  |  |  |  |  |  |  |  |  |  |
| 3 | T3N2aM0 | IIIB | *JAK3* | 17945643 | C>T | Splice site | - | 664 | 180 | 27.11 | 0 | 0 | 0 | 0 | 0 | 0 |
|  |  |  | *TP53* | 7577121 | G>A | R273C | COSM10659 | 553 | 73 | 13.20 | 437 | 0 | 0 | 615 | 0 | 0 |
|  |  |  | *NOTCH1* | 139399386 | C>T | R1586H | - | 587 | 76 | 12.95 | 0 | 0 | 0 | 0 | 0 | 0 |

Abbreviations: var. cov., variant coverage; var. freq., variant frequency.

^a^TNM Classification of Malignant Tumors, 7th Edition

bStop codon
