# Supplementary material for: Individualized Mutation Detection in Circulating Tumor DNA for Monitoring Colorectal Tumor Burden Using a Cancer-Associated Gene Sequencing Panel
Source: PLoS One. 2016 Jan 4;11(1):e0146275. doi: 10.1371/journal.pone.0146275 (PMC4699643; doi:10.1371/journal.pone.0146275)
Supplement: S5 Table — (DOCX) [file pone.0146275.s011.docx]

**S5 Table.** Tumor and ctDNA concentrations of validated marker mutations in 19 cases by ddPCR

| Case | TNM^b^ | pStage^b^ | Gene | Position | Nucleotide mutation | Amino Acid Change | COSMIC ID | Tumor | | | Pre-operation in cfDNA | | | Post-operation in cfDNA | | | |
| --- | --- | --- | --- | --- | --- | --- | --- | --- | --- | --- | --- | --- | --- | --- | --- | --- | --- |
|  |  |  |  |  |  |  |  | WT conc.^a^ | MT conc.^a^ | MT ratio^a^ | WT conc.^a^ | MT conc.^a^ | MT ratio^a^ | WT conc.^a^ | MT conc.^a^ | MT ratio^a^ |  |
| 4 | T3N0M0 | IIA | *BRAF* | 140453136 | A>T | V600E | COSM33765 | 129 | 52.5 | 28.9 | 10.7 | 0 | 0 | 60.4 | 0 | 0 | |
|  |  |  | *AKT1* | 105246551 | C>T | E17K | COSM33765 | 342 | 145.9 | 29.9 | 24.7 | 0.16 | 0.64 | 128 | 0 | 0 | |
|  |  |  |  |  |  |  |  |  |  |  |  |  |  |  |  |  | |
| 5 | T3N1bM0 | IIIB | *TP53* | 7578406 | C>T | R175H | COSM10648 | 908 | 312 | 25.6 | 38.8 | 0.44 | 1.12 | 149 | 0.15 | 0.10 | |
|  |  |  |  |  |  |  | COSM99914 |  |  |  |  |  |  |  |  |  | |
|  |  |  |  |  |  |  | COSM99022 |  |  |  |  |  |  |  |  |  | |
|  |  |  | *KRAS* | 25398284 | C>T | G12D | COSM521 | 444 | 114 | 20.4 | 17.9 | 0.15 | 0.82 | 68.5 | 0.04 | 0.06 | |
|  |  |  |  |  |  |  |  |  |  |  |  |  |  |  |  |  | |
| 6 | T3N1aM0 | IIIB | *TP53* | 7577120 | C>T | R273H | COSM10660 | 23.3 | 16.2 | 41.0 | 20.5 | 0.09 | 0.44 | 28.2 | 0.04 | 0.14 | |
|  |  |  |  |  |  |  | COSM99729 |  |  |  |  |  |  |  |  |  | |
|  |  |  | *KRAS* | 25398284 | C>T | G12D | COSM521 | 20.4 | 6.8 | 24.9 | 12.3 | 0.09 | 0.70 | 16.7 | 0 | 0 | |
|  |  |  |  |  |  |  |  |  |  |  |  |  |  |  |  |  | |
| 7 | T3N0M0 | IIA | *KRAS* | 25398285 | C>A | G12C | COSM516 | 182 | 75.3 | 29.3 | 25.1 | 0 | 0 | 68.6 | 0.04 | 0.07 | |
|  |  |  |  |  |  |  |  |  |  |  |  |  |  |  |  |  | |
| 8 | T2N0M0 | I | *TP53* | 7577121 | G>A | R273C | COSM10659 | 384 | 114.2 | 22.9 | 17.7 | 0 | 0 | 200 | 0.21 | 0.10 | |
|  |  |  |  |  |  |  | COSM99933 |  |  |  |  |  |  |  |  |  | |
|  |  |  |  |  |  |  |  |  |  |  |  |  |  |  |  |  | |
| 9 | T3N1aM0 | IIIB | *ERBB2* | 37880220 | A>G | L755S | COSM14060 | 807 | 288 | 26.3 | 411 | 0.07 | 0.02 | 581 | 0.18 | 0.03 | |
|  |  |  |  |  |  |  |  |  |  |  |  |  |  |  |  |  | |
| 10 | T2N1aM0 | IIIA | *APC* | 112175216 | G>T | E1309^c^ | COSM18775 | 3160 | 396 | 11.1 | 845 | 1.03 | 0.12 | 752 | 0.77 | 0.10 | |
|  |  |  |  |  |  |  |  |  |  |  |  |  |  |  |  |  | |
| 11 | T3N1bM0 | IIIB | *TP53* | 7577538 | C>T | R248Q | COSM99020 | 979 | 182.1 | 15.7 | 143 | 0.31 | 0.22 | 523 | 0.15 | 0.03 | |
|  |  |  |  |  |  |  | COSM99602 |  |  |  |  |  |  |  |  |  | |
|  |  |  |  |  |  |  | COSM10662 |  |  |  |  |  |  |  |  |  | |
|  |  |  |  |  |  |  |  |  |  |  |  |  |  |  |  |  | |
| 12 | T3N1bM0 | IIIB | *KRAS* | 25398281 | C>T | G13D | 1320 | 1320 | 11.5 | 7.8 | 74.5 | 0 | 0 | 226 | 0 | 0 | |
|  |  |  |  |  |  |  |  |  |  |  |  |  |  |  |  |  | |
| 13 | T2N0M0 | I | *TP53* | 7578268 | A>C | L194R | 1726 | 1726 | 473 | 21.5 | 161 | 0 | 0 | 446 | 0.07 | 0.02 | |
|  |  |  |  |  |  |  |  |  |  |  |  |  |  |  |  |  | |
| 14 | T3N1bM0 | IIIB | *KRAS* | 25398284 | G>T | G12V | 565 | 565 | 278 | 33.0 | 183 | 0 | 0 | - | - | 0 | |
|  |  |  |  |  |  |  |  |  |  |  |  |  |  |  |  |  | |
| 17 | T3N1bM0 | IIIB | *TP53* | 7577568 | C>T | C238Y | 1893 | 1893 | 439 | 18.8 | 15.2 | 0 | 0 | 56.6 | 0 | 0 | |
|  |  |  |  |  |  |  |  |  |  |  |  |  |  |  |  |  | |
| 19 | T2N0M0 | I | *TP53* | 7578404 | A>T | C176S | 1901 | 1901 | 756 | 28.5 | 395 | 0.18 | 0.05 | 233 | 0.22 | 0.09 | |
|  |  |  | *PIK3CA* | 178936091 | G>A | E545K | COSM763 | 488 | 199 | 29.0 | 333 | 0.12 | 0.04 | 222 | 0.30 | 0.13 | |
|  |  |  |  |  |  |  |  |  |  |  |  |  |  |  |  |  | |
| 20 | T3N1bM0 | IIIB | *BRAF* | 140453136 | A>T | V600E | COSM476 | 251 | 76.1 | 23.3 | 59.3 | 0 | 0 | 87.7 | 0 | 0 | |
|  |  |  |  |  |  |  |  |  |  |  |  |  |  |  |  |  | |
| 21 | T3N0M0 | IIA | *KRAS* | 25398284 | G>T | G12V | COSM520 | 453 | 279 | 38.1 | 27.3 | 0.04 | 0.15 | 56.1 | 0.03 | 0.05 | |
|  |  |  |  |  |  |  |  |  |  |  |  |  |  |  |  |  | |
| 22 | Adenoma | - | *GNAS* | 57484421 | G>A | R201H | COSM27895 | 1645 | 836 | 33.7 | 455 | 0.12 | 0.03 | 84.9 | 0.06 | 0.07 | |
|  |  |  | *BRAF* | 140453136 | A>T | V600E | COSM476 | 778 | 362 | 27.5 | 203 | 0 | 0 | 41.3 | 0 | 0 | |
|  |  |  |  |  |  |  |  |  |  |  |  |  |  |  |  |  | |
| 24 | Adenoma | - | *KRAS* | 25398284 | C>T | G12D | COSM521 | 797.5 | 327 | 29.1 | 143 | 0 | 0 | 9.6 | 0 | 0 | |
|  |  |  |  |  |  |  |  |  |  |  |  |  |  |  |  |  | |
| 25 | T1NXMX | NA^a^ | *KRAS* | 25398284 | C>A | G12V | COSM520 | 873 | 10.5 | 1.2 | 15.1 | 0 | 0 | 22 | 0.03 | 0.14 | |
|  |  |  |  |  |  |  |  |  |  |  |  |  |  |  |  |  | |
| 27 | Adenoma | - | *KRAS* | 25398284 | C>T | G12D | COSM521 | 446 | 19 | 4.1 | 11.9 | 0 | 0 | 17.7 | 0 | 0 | |

^a^Concentrations (copies/μl) were calculated based on the number of positive and negative droplets from the specific probe. The average number (±SD) of droplets in tumor (10968 ± 4218), Pre-operation (12010 ± 2868), and post-operation (12004 ± 3736).

Abbreviations: WT conc., Wild Type concentration; MT conc., Mutation Type concentration; MT ratio, Mutation ratio; NA, Not Applicable.

^b^TNM Classification of Malignant Tumors, 7th Edition

^c^Stop codon
